# Current Understanding of Myelomatous Mesenchymal Stromal Cells Extended through Advances in Experimental Methods

**DOI:** 10.3390/cancers13010025

**Published:** 2020-12-23

**Authors:** Michiko Ichii, Naoki Hosen

**Affiliations:** Department of Hematology and Oncology, Osaka University Graduate School of Medicine, Osaka 5650871, Japan; hnaoki@bldon.med.osaka-u.ac.jp

**Keywords:** mesenchymal stromal cells, experimental models, multiple myeloma

## Abstract

**Simple Summary:**

As the amount of information available has grown, now it is known that many types of non-hematopoietic cells, including mesenchymal stem/progenitor cells, mature mesenchymal cells, and endothelial cells, as well as mature hematopoietic cells such as monocytes, macrophages, T-cells, and B-cells, have roles in the pathogenesis of multiple myeloma. This review focuses on the role of mesenchymal cells in the microenvironment of multiple myeloma. We summarize the experimental strategies and current understanding of the biological roles in the pathogenesis of myeloma. Furthermore, we discuss the possible clinical applications targeting mesenchymal cells.

**Abstract:**

Multiple myeloma is an incurable cancer formed by malignant plasma cells. For the proliferation and survival of myeloma cells, as well as the occurrence of the complications, numerous intra- and extra-cellular mechanisms are involved. The interaction of myeloma cells with the microenvironment is known to be one of the most critical mechanisms. A specific microenvironment could affect the progression and growth of tumor cells, as well as drug resistance. Among various microenvironment components, such as hematological and non-hematological cells, and soluble factors (cytokines, chemokines, and extracellular matrix (ECM) proteins), in this review, we focus on the role of mesenchymal cells. We aimed to summarize the experimental strategies used for conducting studies and current understanding of the biological roles in the pathogenesis of myeloma. Furthermore, we discuss the possible clinical applications targeting mesenchymal cells.

## 1. Introduction

Multiple myeloma (MM) is a malignant plasma cell (PC) disease that infiltrates the bone marrow (BM). One of the characteristics of MM is the occurrence of various symptoms and signs, such as hypercalcemia, renal dysfunction, anemia, and bone lesions, with the progression of disease [1,2]. Some of these symptoms are induced upon accumulation or hyperviscosity of monoclonal proteins produced by MM cells. Many types of soluble factors secreted by MM cells and direct and indirect crosstalk between myeloma cells and other cells affect hematopoiesis, osteoclastogenesis, angiogenesis, and osteogenesis in BM. MM is also characterized by the premalignant condition, monoclonal gammopathy of undetermined significance (MGUS), which occurs before the onset of MM. Chromosomal translocations involving 14q and hyperdiploidity are generated as an initial step of transformation to MGUS in post-germinal center B-lymphocytes or immunoglobulin-producing PCs, and then the transformed cells migrate to BM. MM develops after the several processes induced by genetic and environmental factors in BM [3,4].

In the last two decades, the development of novel agents, such as immunomodulatory drugs, proteasome inhibitors, monoclonal antibodies, and histone deacetylase inhibitors (HDACis), as well as the high-dose chemotherapy, followed by autologous stem cell transplantation, dramatically improved patients’ outcomes [5,6]. However, despite the high response rates to the initial therapy, almost all patients develop drug resistance over time, and MM is still considered to be incurable. For the migration of MM cells to BM and following the multistep transformation to malignant diseases, numerous intra- and extra- cellular mechanisms are involved. The interaction with the microenvironment is known to be one of the most important mechanisms for the progression and growth of tumor cells, as well as the development of drug resistance. In this review, we focus on the role of mesenchymal cells as a cellular component of the MM microenvironment. After summarizing the constituents of the BM microenvironment, we further discuss the experimental strategies for studies, knowledge from these experiments, and the possible clinical applications targeting mesenchymal cells.

## 2. Hematopoietic Microenvironment in BM

### 2.1. Microenvironment for Hematopoietic Stem Cells or Plasma Cells in Normal BM

Based on the hypothesis that cells surrounding hematopoietic stem cells (HSCs) determine the behavior of HSCs in BM, the effects of cellular components on HSC population have been extensively evaluated [7,8,9,10,11]. As the information available has expanded enormously, now it is known that many types of non-hematopoietic cells, including mesenchymal stem/progenitor cells, mature mesenchymal cells, endothelial cells, sympathetic neurons, non-myelinating Schwann cells, perivascular cells, and mesodermal derived cells, as well as mature hematopoietic cells such as monocytes, macrophages, regulatory T-cells, neutrophils, and megakaryocytes, exhibit crucial roles in the maintenance of the stemness of HSCs and the coordinated production of different types of hematopoietic cells [9,10,12]. These cellular components are involved in hematopoiesis through the direct interaction with HSCs, and also in the indirect ways through the secretion of soluble factors (cytokines, chemokines, and extracellular matrix (ECM) proteins) and small vesicles of exosomes packaging microRNAs (miRNAs), and the regulation of other microenvironmental cells or factors.

One of the well-studied subjects regarding the microenvironment in BM is mesenchymal lineage. Mature mesenchymal cells include bone, cartilage, fat, and muscle cells. Mesenchymal stromal cells (MSCs) with the ability to perform multi-lineage differentiation, self-renewal, and reconstitution in vivo are detected in several organs, such as BM, adipose tissue, connective tissue, umbilical cord, and placental tissue [8,11]. In BM cavity, a range of adipo- and osteo-lineage cells are found to exist at different developmental stages, including the most primitive MSCs and several distinct stages of lineage-committed progenitors, to terminally differentiated cells. In vivo experiments using transgenic mouse labeled with Nestin, leptin-receptor, or CXC chemokine ligand (CXCL), 12 have been useful to determine the critical roles of MSCs located in the perivascular area in HSC maintenance, and that the osteoprogenitors support B lymphopoiesis [11,13,14,15,16,17].

Regarding the microenvironment of plasma cells, it is reported that plasmablasts, which express high levels of CXC chemokine receptor (CXCR) 4, migrate and home to BM, where they are retained by CXCL12-expressing stromal cells, from secondary lymphoid organs [17,18,19,20,21]. Other chemokines, including CXCL9, CXCL10, and CXCL11 in mice, which are the ligands of CXCR3, and CXCL 16 in human, which is the ligand of CXCR6, are also involved in the entry of plasmablasts into BM [22,23,24]. Direct adhesion signals between PCs and stromal cells, including very late antigen 4 (VLA-4), a dimer of integrin α4 and β1, and the receptor for vascular cell adhesion molecule 1 (VCAM-1) and fibronectin, and the lymphocyte function associated antigen 1 (LFA-1), a dimer of integrin αL and β2, and the receptor for intercellular adhesion molecule 1 (ICAM-1), are essential [17,18,19,25,26]. For the survival of PCs, cytokines, including a proliferation-inducing ligand (APRIL) and B-cell activating factor (BAFF), members of tumor necrosis factor (TNF) superfamily, and IL-6, are critical [21,22,27,28]. Among cells which secret APRIL, such as monocytes, macrophages, eosinophils, osteoclasts, and fibroblasts, eosinophils have been shown to play the important role as the PC microenvironment in mice [29]. Plasma cells in BM were localized together with eosinophils, and the eosinophil-deleted mice exhibited a decrease in the number of long-lived PCs in BM with migration of PCs to spleen. For the survival of long-lived plasma cells, the retention with CXCL12 is also known to be important [14,17,22,26]. Tokoyoda et al. reported that plasma cells in murine BM were located in contact with CXCL12-abundant reticular cells, and CXCR4-deletion in B-lineage cells relocated PCs from BM to spleen, indicating an essential role of mesenchymal cells as the cellular component of PC microenvironment [17].

### 2.2. Microenvironment for Malignant Cells in BM

In hematological malignancies, the effects of the microenvironment are higher than previously thought [9,30,31,32]. Neoplastic cells interact with almost all of the cellular components in BM, such as endothelial, mesenchymal, and hematopoietic cells, inducing a favorable environment for their survival. Leukemic cells are involved in the secretion of various types of soluble factors and the differentiation of MSCs [33,34,35,36]. Dissemination of malignant cells changes the oxygen concentration in BM and enhances angiogenesis via increasing the levels of hypoxia-inducible factors (HIFs), angiopoietin and proangiogenic cytokines [30,32]. The tumor-specific microenvironment affects the survival and drug resistance of tumor cells [31]. For example, recent studies have reported that, in mice with myeloproliferative neoplasms, MSCs in BM are essential for tumor proliferation and creation of the fibrotic environment [37,38]. Furthermore, the tumor microenvironment suppresses the immune system through the direct activation of regulatory T-cells, inhibition of B-cell proliferation, and cytokine secretion [32,39,40]. Intriguingly, several studies proved that the abnormal BM microenvironment could lead to the development of hematological malignancies [9,38,41,42,43,44]. With the deletion of *Dicer-1* in murine osteoprogenitors, normal HSCs were transformed into the myelodysplasia, which subsequently developed acute myeloid leukemia [41]. Kode et al. reported that constitutive activation of β-catenin in osteoblast induced leukemogenic transformation in mice [43,44].

Multiple myeloma cells, which reside in BM, in most cases, are highly dependent on the BM microenvironment, especially mesenchymal cells. In the following section, we focus on the studies investigating the role of mesenchymal cells in MM with different points of view.

## 3. The Role of Mesenchymal Cells in Myeloma Microenvironment

**Figure 1:**
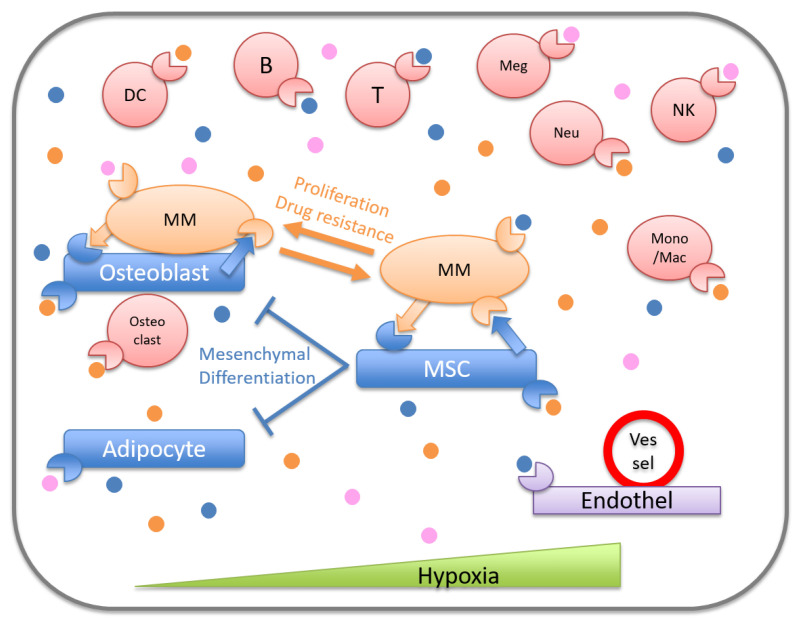
Bone marrow microenvironment of multiple myeloma. In bone marrow, myeloma cells and the surrounding cells interact with each other in direct or indirect ways, inducing a favorable environment for the survival of multiple myeloma. MM, multiple myeloma; MSC, mesenchymal stromal cell; Endothel, endothelial cell; Mono/Mac, monocyte or macrophage; NK, natural killer cell; Neu, neutrophil; Meg, Megakaryocyte; DC, dendritic cell; ●, soluble factors.

The crosstalk between MM cells and other cell types in BM supports the dissemination, survival, migration, transformation, drug resistance, and relapse of MM cells [32,45,46]. MM cells and the surrounding cells adapt to the microenvironmental condition to favor MM maintenance through altering the concentrations of soluble factors and oxygen, and enhancing angiogenesis and hypoxia [47,48,49]. Hematopoiesis, osteogenesis, adipogenesis, and immunity are also affected in the MM microenvironment (Figure 1).

There are various ways for studying the pathogenesis of MM microenvironment. In this section, we discuss the results obtained from using different types of approaches for the research about the role of mesenchymal cells as the MM microenvironment. Like other cancer types, MM has special properties favoring survival. Additionally, MM develops from the precursor state of MGUS, and there are many steps underlying it until the final evolution to extramedullary diseases [50]. The treatment of MM patients after the diagnosis sometimes continues over decades. In studies on MM, depending on the objective of the research, the experimental strategies and subjects are decided to clarify what is really happening in patients, and some of technical problems remain unresolved.

### 3.1. Studies with Myeloma Cells

The environmental influences are traceable through analyzing MM cells, and MM cells also impact MSCs. Studies using tumor cells have shown that MM cells secret the inhibitory factors for osteoblastogenesis, such as hepatocyte growth factor (HGF) and TNFα [49,51]. Inhibitors of canonical Wnt signaling, including Dickkopf (DKK)1, secreted frizzled related protein (sFRP) −2 and −3, and sclerostin, are also produced by MM cells, leading the suppression of osteoblast formation [52,53,54,55]. Activated signaling of basic fibroblast growth factor (bFGF)1 and TNFα, secreted by MM cells, stimulates IL-6 production in MSCs [49,56].

The results of several studies with MM cells indicate that they interact with the surrounding cells via the adjacent and direct contact. Chemokines, such as CC chemokine ligand (CCL) 25, produced by MM cells, and CXCR4 expressed on the surface of MM cells, lead to the migration of MM cells close to MSCs [32,48,57,58]. Cell adhesion molecules, including integrins, cadherins, and selectins, are also important for the direct interaction between MM cells and MSCs [59,60,61,62,63,64,65,66,67,68]. Overexpression of c-MAF, which is observed in half of patients with MM, increases integrin β7 at both RNA and protein levels in MM cells, and this molecule induces the adhesion of MM cells to MSCs, thereby leading the induction of vascular endothelial growth factor (VEGF) [69,70]. Another report indicated that the interaction of integrin α4β7, with its counter-receptor, mucosal addressin cell adhesion molecule (MADCAM)-1, contributed to the retention of MM cells in BM [64]. Recently, we identified the activated form of integrin β7 as a MM-specific marker through screening the monoclonal antibodies reacting with MM cell lines [65]. In our observation, MM cells derived from approximately 90% of patients (45 of 51 samples) expressed active integrin β7, while integrin β7 expressed on T- and B-cells mainly corresponded to the resting structure. Although it is known that large conformational changes are required for activating integrins, the detailed mechanisms in MM remain unknown.

Using multicolor flow-cytometry technique, Paiva et al. showed that the residual disease clones after treatment exhibited high expression of integrins, such as integrin α4, α5, and β1, as well as CXCR4, compared to MM cells isolated before the treatment [66,67]. These results indicated that residual MM cells directly interact with the special microenvironment and, thus, could be protected from chemotherapy. Loss of activated leukocyte cell adhesion molecule (ALCAM) expression on residual MM cells was involved in chemoresistance; however, the function of ALCAM in the interaction with the microenvironment is not yet fully understood.

### 3.2. Studies with Human MSCs

The experiments using MSCs isolated from MM patients’ BM (MM-MSCs) have provided direct evidence for the important roles of the mesenchymal cells in MM pathogenesis and the interaction between MM cells and MSCs. MM-MSCs are different from those derived from normal healthy donors (ND-MSCs), and these alternations are not caused by normal responses to osteolysis induced by activated osteoclasts [32,45,46,71,72]. Due to the difficulties in the isolating MSCs from BM, studies on MM-MSC were limited for a long time.

In 2000s, genetic analyses with MM-MSCs demonstrated that transcriptomes, such as *IL-6, DKK1* and *growth differentiation factor (GDF)15*, which regulate angiogenesis, osteogenic differentiation, and tumor growth, were distinctively expressed in MM-MSCs, as compared to ND-MSCs [73,74]. Another study using bioinformatics analyses showed that the genes associated with cell cycle, immune response, and bone metabolism were significantly regulated [75,76,77]. In vitro experiments with a co-culture system indicated that the supportive capacity of T-cell proliferation was impaired with high expression of IL-6 by MM-MSCs [78,79]. The proliferation capacity of MSCs was inhibited by reduced expression of the receptors for platelet-derived growth factor (PDGF)α, PDGFβ, insulin-like growth factor (IGF) 1, epidermal growth factor, and bFGF on MM-MSCs, while others reported a comparable expansion between MM-MSCs and HD-MSCs [73,78,79,80,81,82]. Other studies have demonstrated the concentration of various soluble factors, such as IL-10, IL-1β, IL-3, TNFα, stem cell factor, granulocyte-macrophage colony-stimulating factor (GM-CSF), HGF, BAFF, decorin, and CCL3, in MM microenvironment are altered by MM-MSCs [75,77,83,84,85,86,87]. For the regulation of cytokine secretion, Li et al. reported that the elongation of telomere length in MM-MSCs affected the expression of *IL-6* and *CCL3* in MM cells [84]. McNee et al. showed that the upregulation of *peptidyl arginine deaminase (PADI)2* mediated IL-6 production in MM-MSCs through the enzymatic deamination of histone H3 arginine 26 to form citrulline [88]. The upregulation of *PADI2* was identified in MSCs derived from both MGUS and MM patients, indicating that the microenvironment is altered at the stage of MGUS. The activation of Notch signaling in MM-MSCs induced by MM cells has also been reported as the mechanism of IL-6 and VEGF production [89].

Increased levels of soluble factors, such as IL-6, BAFF, and GDF15, and a decreased level of decorin in BM plasma, promote the proliferation of MM cells [73,74,85,90]. In addition, recent studies showed that MM-MSCs secreted exosomes and microvesicles, which are transferred to MM cells [91,92]. The exosomes derived from MM-MSCs included oncogenic proteins, cytokines, and adhesion molecules and promoted MM proliferation, as compared to those derived from HD-MSCs [91]. Specifically, the lower levels of *miRNA-15a*, a tumor suppressor, and higher expression of *miRNA-10a* in these vesicles were identified [91,92]. While transferred *miRNA-10a* enhanced the proliferation of MM cells, the accumulation in MM-MSCs inhibited the proliferation of MSCs with the induced apoptosis, suggesting the therapeutic possibility of the transfer inhibition of MM-MSC-derived exosomes [92].

Using culture experiments, which are helpful for analyzing functions and interactions of specific cell subsets or molecules, the importance of interaction with CXCL12, VCAM-1, and ECM proteins has been revealed [63,93,94]. Another interesting study performed by Kikuchi et al. showed that stromal cell adhesion-dependent epigenetic alternation in MM cells contributes to drug resistance [95,96]. They demonstrated that inactivation of enhancer of zeste homolog (EZH) 2 by MM-MSCs reduced the abundance of H3K27me3 and altered the expression of several genes in MM cells. The direct interaction between MSCs and MM cells blocked H3K27 hypermethylation induced by cytotoxic anti-cancer agents, such as doxorubicin and melphalan, and maintained the expression of anti-apoptotic genes of *BCL2, IGF1, and HIF1a*, leading to the drug-resistant survival of MM cells. The indirect effect of MM-MSCs with the secreted soluble factors on upregulation of *KDM6B* gene, which removes H3K27 di- and tri-methylation in MM cells, has also been reported [97].

The abnormalities of mesenchymal cells are also involved in the bone complication in MM together with the activation of osteoclast [82,98,99]. MM-MSCs derived from patients with bone lesions showed distinct gene expression profiles, compared to those from patient without bone complications [86]. The differentiation capacity of MM-MSCs to adipocytes and osteoblasts is suppressed [78,79,100]. Circulating cytokines, such as transforming growth factor (TGF)β, TNFα, interferon (IFN)γ, IL-1β, and IL-6, as well as the direct interaction with MM cells, promote the apoptosis of osteoprogenitors [89,101,102,103]. Inhibitors of the canonical Wnt signaling pathway produced by MM cells and altered expression of miRNA, such as *miRNA-138* and *-223* in MM-MSCs, are also shown to impair osteoblast differentiation [52,80,89,104].

The underlying mechanisms of the transformation of HD-MSCs to MM-MSCs are partially understood. As mentioned above, soluble factors in BM influence the differentiation of MSCs. Exposure to MM cells also induces the special MSC phenotypes. Xu et al. reported that *miRNA-135b*, which was aberrantly expressed in MM-MSCs and inhibited the osteogenesis, was upregulated in ND-MSCs co-cultured with MM cells, while the expression recapitulated to the normal level after removing MM cells from the culture [105]. Interestingly, there is evidence indicating that MM-MSCs remain dysfunctional without the coexistence of MM cells, for example, after the achievement of complete remission in MM patients [45,78]. MM-MSCs cultured without MM cells demonstrated overproduction of cytokines, such as IL-6 and GDF15, thereby favoring growth of MM cells [73]. MM-MSCs possess genomic alternations with a specific pattern of gene region, and aberrant methylation status in MM-MSCs has also been reported [106,107,108]. Adamik et al. demonstrated that heterochromatin silencing of the promoter of *Runt-related transcription factor (RUNX)2* prolongs impaired osteogenesis [109]. The transcriptional repressor of *RUNX2*, *Gfi1*, which is upregulated in MM-MSC by TNFα or IL-7 during MM progression, recruits *HDAC1* and *EZH2*, and increases H3K27me3 on *RUNX2* [109,110].

In many studies about human MSCs, adhesion methods for isolation have been commonly used, considering the difficulties in direct isolation of MSCs from BM and the proliferation capacity in vitro [8]. While macrophages and other non-mesenchymal cells are often contaminated in the adhesion methods with murine BM, mesenchymal lineage cells are efficiently cultivated in humans. The International Society for Cellular Therapy (ISCT) issued the minimal criteria for defining MSCs in BM [111]. According to the recommendation from ISCT, the cells adhering to plastic after culturing isolated marrow cells, which lack expression of CD45, CD34, CD14, CD11b, CD79a, or CD19, but express CD105, CD73, and CD90, and are capable of differentiating into osteogenic, adipogenic, and chondrogenic lineages, should be considered as MSCs. On the other hand, recent technical advances in flow cytometry have enabled the isolation of MSCs, which express several surface markers, such as CD271, CD146, SSEA4, CD49a, and PDGF receptors in human, without maintaining cell cultures. It has been reported that there are differences between freshly isolated and cultured MSCs in terms of expression of RNAs, cell surface molecules, and the reconstitution capacity in vivo [76,112,113,114,115]. Although the experimental results mentioned above were acquired from studies with MSCs derived from patients, it is possible that MSCs isolation method might affect the results. This needs to be considered especially for translating them into therapeutic applications.

### 3.3. Studies with Mouse Models

The concept of microenvironment explains that the neighboring subsets of cells and extracellular substrates regulate the survival and function of MM cells in the region where MM cells reside. Ex vivo studies using the cells isolated from patients have limitations on the anatomical evaluation, and the condition in culture experiments cannot recapitulates the MM microenvironment in BM. Mouse models are useful tools for understanding the interaction between MM cells and the surrounding microenvironment.

Mouse strain C57BL/KalwRij is known to spontaneously develop monoclonal disorder of immunoglobulin-producing B-cells, resembling MGUS in human [116,117,118]. Although the probability of progression to MM and Waldenstrom Macroglobulinemia is less than 1% in primary mice, the recipient mice transplanted with primary cells or the established 5T cell lines develop MM with bone lytic lesions. Using this 5T mouse model, Fowler et al. reported that adiponectin secreted from MM-MSCs decreased with the progression of disease after 5T cell transplantation, and a similar phenomenon was observed in MGUS patients who subsequently developed MM [119]. Although adiponectin was originally identified as a protein secreted from adipose tissue, it has since been recognized that adiponectin is also secreted by a range of cell types, including MSCs in BM. Adiponectin induced MM cell apoptosis, and its therapeutic effects were shown in the study. Using this model, they also demonstrated the upregulated DKK1 secretion from MM-MSCs and the critical role of BMP signaling in bone destruction induced by MM [120,121]. Intriguingly, taking advantage of mouse experiments, Lawson et al. dissected the microenvironments for two types of MM cell fractions with distinct cell-cycle status [122]. They visualized eGFP-labeled 5TGM1 MM cells in real time, using intravital two-photon microscopy, and showed that MM cells colonized in the endosteal area after transplantation. Furthermore, dormant MM cells, which highly expressed *VCAM-1* and *AXL*, were retained in direct contact with bone-lining osteoblasts. Conversely, actively dividing MM cells were found at locations distant from the bone surface, and bone resorption induced by osteoclast activation released dormant MM cells from their own microenvironment, thereby entering into the cell cycle. They also demonstrated that the dormancy in MM cells was associated with drug resistance, using melphalan-treated mice. In addition, the distinct transcriptome profile of the dormant MM cells was induced by cocultures with osteoblastic cells [123].

Several types of transgenic (Tg) mice, in which the expression of *MAF*, *XBP-1*, or *MYC* genes is enforced in the B-cell compartment, develop MGUS and MM [124,125,126]. Among them, Vk*MYC Tg mice, in which the activation of *MYC* is under the control of *Ig kappa light chain* gene regulatory elements, show anemia, osteolytic bone lesion, and kidney damage, resembling human MM symptoms, and the MM cells are transplantable with the reconstitution in BM [126]. This suggests a potential application of studying the microenvironment. Using this mouse model, the detailed mechanisms of immunomodulation, anemia, and hypoxic condition in MM-specific microenvironment have been revealed [47,127,128,129].

### 3.4. Studies with Humanized Mice

There are species differences of MM cells and MSCs between human and mice. To evaluate the interaction of human MM cells with the microenvironment in vivo, the immunodeficient mice, such as severe combined immunodeficiency (SCID) and non-obese diabetic (NOD)/SCID mice, were first used [116,130,131]. Subsequently, the introduction of intratibial injection methods, as well as the development of NOD/SCID-based highly immunodeficient mice, such as NOG (NOD/*Shi*-SCID/*IL2Rγ*^−/−^), and NSG (NOD/*LtSz*-SCID/*IL2Rγ*^−/−^), and *Rag2*^−/−^ *IL2Rγ*^−/−^ mice, has led to the improvement in the ability of BM engraftment of patient MM cells [132,133,134,135]. In these humanized models, MM cells labeled with bioluminescent and fluorescent reporters can be tracked by using MRI, PET, and micro CT imaging [132,136]. MM cells in SCID mice disseminate in various organs, such as the spleen, liver, lungs, and BM, after intravenous injection, while subcutaneous injection causes palpable tumors with growth at injection site. Only a few types of human cell lines, such as KMS12-BM and U266, reconstitute dominantly in BM after injection into the immunodeficient mice [116,132]. Using these mouse models, in which the bone lytic lesion develops with the MM cell line infiltration in mice BM, the inhibitory effect of neutralizing human IL-6 receptor on MM proliferation, and the co-localization of human MM cells in endosteal area have been shown [132,137]. In some studies, to assess the role and therapeutic possibility, co-injection of MSCs producing soluble factors, such as IFN and osteoprotegerin, was conducted [131,138].

Recently, mice expressing human genes were developed to mimic human BM environment [139,140]. Humanization of *IL-6*, along with *macrophage-colony-stimulating factor, IL-3, GM-CSF, thrombopoietin*, and *signal regulatory protein-α*, resulted in the enhancement of patient MM cell-reconstitution in recipient BM [139]. No improvement of PC engraftment was observed in immunodeficient mice with human *BAFF* expression [140]. These novel techniques of mouse humanization would be useful for studying the MM microenvironment.

To reproduce human microenvironment, a distinct type of mouse model, which is known as the SCID-hu model, has been established [136,141,142]. In this model, MM cells, such as IL-6-dependent INA-6 MM cell line and primary patient MM cells, were injected into a human bone chip previously implanted in the flank of mice, and the successful engraftment in human BM was observed. This model provided the evidence of the requirement of IL-6 and human specific BM microenvironment, and a useful tool for evaluating the effects of drugs [119,136]. Using the SCID-hu model, osteoblast suppression through Ephrin B2/B4 interaction was described [143]. However, this model has several limitations on the restricted availability of human fetal bones and different nature between fetus and adult. Although rabbit bone is sometimes used for the replacement of fetal bone chip, the problems remain unresolved [135,144]. To overcome the limitations of SCID-hu models, fetal bone chip can be replaced by using an adult BM ossicle with three-dimensional scaffold, and work on technical improvements is currently in progress [145,146,147].

## 4. Therapeutic Application Targeting the Effects of MM-MSCs

Several studies focusing on MM-MSCs have revealed the important roles in MM pathogenesis, which include (i) the supportive effects on MM proliferation, (ii) the suppression of osteoblastogenesis from MSCs, (iii) the induction of drug resistance and dormancy in MM cells, and (iv) the immunosuppressive effects (Table 1). Some of the experimental methods, such as cultures and humanized mouse models, have been useful in predicting clinical efficacies and have shown that the inhibition of pathways in MSCs activated by MM could be promising candidates for the therapeutic target. Here, we explain the results of preclinical and clinical studies on agents with high potential for clinical application (Table 2).

MM-MSCs support the proliferation of MM cells with the production of cytokines, such as IL-6 and BAFF, transfer of exosomes into MM cells, and the direct interaction. The anti-IL-6 chimeric antibody, siltuximab has been tested in MM patients [148,149,150]. While treatment with siltuximab did not improve the outcome in studies of newly diagnosed and relapsed/refractory patients, another clinical study showed the inhibition tended to delay the progression of high-risk smoldering MM. Preclinical studies using cultures and humanized mice showed the inhibitors of JAK signaling, such as tofacitinib and ruxolitinib, were effective in suppressing MM cell proliferation [151,152]. Umezu et al., reported the inhibitory effects of the extracellular vesicle release inhibitor, FTY720, which has been approved for the treatment of multiple sclerosis in US and Europe, on MM growth in vitro and in vivo [92].

The capacity of MM-MSCs to differentiate osteoblasts is suppressed by the increased levels of cytokines, such as Wnt signaling inhibitors, TGFβ, and IL-6, as well as cell-intrinsic pathways. In clinical trials with DKK1 inhibitor, BHQ880, and TGFβ antagonists, such as sotatercept and luspatercept, significant improvements in bone complications have not been reported so far [153,154,166]. Considering the suppression of osteogenic genes with aberrant methylation status in MM-MSCs, epigenetic inhibitors, such as DNA methyltransferase inhibitors (DNMTis) and HDACis, which are used for the various types of cancer therapies, might be effective [108,109]. Knowledge about the effects of DNMTi on MSCs is limited [155]. Preclinical studies have shown that some HDACis, such as trichostain A, valproic acid, and sodium butyrate, promote osteoblastogenesis from MSCs, and the effects of vorinostat are controversial [156,157,158,159,160]. Clinical observations, as well as preclinical studies, have demonstrated that bortezomib, a proteasome inhibitor, which is commonly used for MM treatment, induces the differentiation to osteoblast [99,161].

The adhesion to mesenchymal cells within the special endosteal microenvironment induces distinct phenotypes characterized by the cell-cycle status and drug resistance of MM cells. Several studies in vitro and in MM mouse models indicate that blocking MM cell adhesion could release MM cells residing in the dormant microenvironment and improve the sensitivity to the treatment. Natalizumab, which has been used for the treatment of multiple sclerosis in clinical practices, prevents the integrin α4-mediated adhesion to their counter-receptors, including VCAM-1 and MADCAM-1. This molecule inhibited the proliferation of MM cells in preclinical studies [162]. Clinical efficacies in MM patients targeting the CXCL12–CXCR4 axis by using plerixafor, olaptesed pegol, and ulocuplumab are currently under investigation [163,164,165].

MM-MSCs exhibit immunosuppressive effects with a decreased ability to support T- and B-lymphocytes [75,78,79]. MM-MSCs regulate both innate and adaptive immune responses through the secretion of IFNγ, IL-1β, IL-6, IL-10, and TNFα [75,83,84]. Considering the recent advances in immune therapy, such as the application of immunocheckpoint inhibitors and chimeric antigen receptor (CAR)-T therapy, the regulation of immune status would be more important in the near future [167,168]. For the treatment of inflammatory diseases and tissue injuries, MSCs derived from healthy donors have already been used in clinical practices [39,169].

## 5. Conclusions and Perspectives

Numerous studies have shown that MM-MSCs are functionally and genetically different from ND-MSCs, leading to supportive characteristics for the migration to BM, proliferation, drug resistance of MM cells, and survival of residual tumor cells. At present, our knowledge regarding the role of MSCs in the emergence of MGUS, progression to MM, and transformation of MM clones during treatment is limited [3,50]. MSCs might affect the etiological events at HSC and B progenitor levels, which contribute to the progression of MM in some patients [170,171]. The effects of MM treatment on MSCs are also largely unknown [76]. In the near future, the improvement in humanized mouse models with advances in gene transduction methods and construction of three-dimensional tissues with scaffold could yield valuable information. Novel techniques to analyze RNA, DNA, and protein expression at the single-cell level, as well as spatial transcriptomic analyses, could help dissect the heterogeneity in MM-MSCs [35,172]. MM is still essentially incurable, and understanding the pathogenesis of MM-MSCs could contribute to developing novel therapeutic strategies and drugs.

## Figures and Tables

**Table 1:** Interaction between multiple myeloma cells and mesenchymal stromal cells.

Cell Type	Effects	Effects	Effects	Regulated Factors	References
MM cells	Effects on MSCs	Soluble factor secretion	Inhibition of osteoblastogenesis	Wnt inhibitors (DKK1, sFRPs, sclerostin), TNFα	[49,51,52,53,54,55]
			IL-6 production in MSCs	bFGF1, TNFα	[49,56]
		Localization with MSCs	Proliferation and drug resistance in MM cells	CCL25, CXCR4 Integrin α4, α5, β1, β7, ALCAM, VCAM-1	[32,48,57,58,59,60,61,62,63,64,65,66,67,68]
			VEGF production in MSCs	Integrin β7	[69]
MSCs	Effects on MM cells	Soluble factor secretion	MM cell proliferation	IL-6, GDF15, BAFF, micro vesicles (miRNA-15a,-10a)	[73,74,83,84,85,86,87,88,89,90,91]
		Localization with MM cells	Proliferation and drug resistance in MM cells	CXCL12, CCL3, ICAM-1, VCAM-1, MADCAM-1	[63,83,84,85,86,87,88,89,90,91,92]
MSCs	Effects on MM microenvironment		Inhibition of osteoblastogenesis	DKK1, TNFα, IL-6, TGFβ	[101,102,103,104]
			immunomodulation	IL-6, IL-10, IL-1β, IL-3, TNFα,	[75,76,77,78,79,83,84,85]
			angiogenesis	VEGF, HGF	[69,89]

MM, multiple myeloma; MSC, mesenchymal stromal cell; DKK1, Dickkopf1; sFRP, secreted frizzled related protein; TNF, tumor necrosis factor; CCL, CC chemokine ligand; CXCR, CXC chemokine receptor; CAM, cell adhesion molecule; ALCAM, activated leukocyte cell adhesion molecule; VCAM, vascular cell adhesion molecule; VEGF, vascular endothelial growth factor; GDF, growth differentiation factor;BAFF, B-cell activating factor; ICAM, intercellular adhesion molecule; MADCAM, mucosal addressin cell adhesion molecule; TGF, transforming growth factor; HGF, hepatocyte growth factor.

**Table 2:** Agents for targeting the effects of MM-MSCs.

Target	Agent	Agent	Agent	Efficacies
MM proliferation	IL-6 chimeric antibody [148,149,150]	Siltuximab	Clinical trials	Not clear
	JAK signal inhibitor [151,152]	Tofacitinib, ruxolitinib	Preclinical studies	Yes
	Microvesicle release inhibitor [92]	FTY720	Preclinical studies	Yes
Osteoblastogenesis suppression	DKK1 inhibitor [153]	BHQ880	Clinical trials	No
	TGFβ antagonist [154]	Sotatercept	Clinical trials	No
	DNMT inhibitor [155]	5-Azacytidine	Preclinical studies	Not clear
	HDAC inhibitor [156,157,158,159,160]	Vorinostat	Preclinical studies	Not clear
	Proteasome inhibitor [161]	Bortezomib	Clinical studies	Yes
Adhesion to MM microenvironment	Integrin α4 inhibition [162]	Natalizumab	Preclinical studies	Yes
	CXCL12-CXCR4 inhibition [162,163,164,165]	Plerixafor, olaptesed pegol, ulocuplumab	Clinical trials	Ongoing

MM, multiple myeloma; MSC, mesenchymal stromal cell; DKK1, Dickkopf1, TGF, transforming growth factor; DNMT, DNA methyltransferase; HDAC, histone deacetylase; CXCL, CXC chemokine ligand; CXCR, CXC chemokine receptor.
